# “A piece of paper is not the same as having someone to talk to”: accessing post-diagnostic dementia care before and since COVID-19 and associated inequalities

**DOI:** 10.1186/s12939-021-01418-1

**Published:** 2021-03-11

**Authors:** Clarissa Giebel, Kerry Hanna, Hilary Tetlow, Kym Ward, Justine Shenton, Jacqueline Cannon, Sarah Butchard, Aravind Komuravelli, Anna Gaughan, Ruth Eley, Carol Rogers, Manoj Rajagopal, Stan Limbert, Steve Callaghan, Rosie Whittington, Lisa Shaw, Mark Gabbay

**Affiliations:** 1 Department of Primary Care & Mental Health, University of Liverpool, Liverpool, UK; 2 NIHR ARC NWC, Liverpool, UK; 3 SURF Liverpool, Liverpool, UK; 4 The Brain Charity, Liverpool, UK; 5 Sefton Older People’s Forum, Liverpool, UK; 6 Wigan Dementia Action Alliance, Wigan, UK; 7 Lewy Body Society, Wigan, UK; 8 Mersey Care NHS Trust, Liverpool, UK; 9 North West Boroughs NHS Trust, Liverpool, UK; 10 Together in Dementia Everyday (TIDE), Liverpool, UK; 11 Liverpool Dementia Action Alliance, Liverpool, UK; 12 House of Memories, Liverpool, UK; 13 Lancashire & South Cumbria NHS Trust, Lancaster, UK; 14 EQE Health, Liverpool, UK; 15 Me2U Daycare, Liverpool, UK; 16 Department of Modern Languages and Cultures, University of Liverpool, Liverpool, UK

## Abstract

**Background:**

Social support services such as day care centres are important in post-diagnostic dementia care to enable people living with dementia stay at home for longer. Little research has addressed potential inequalities in access, with no research on variations before and since COVID-19. The aim of this study was to explore inequalities in social support service usage before and since the pandemic.

**Methods:**

Unpaid carers and people living with dementia were interviewed over the phone about their experiences of accessing social support services before and since the COVID-19 pandemic. Transcripts were analysed for key themes using inductive and deductive thematic analysis.

**Results:**

Fifty participants (42 unpaid carers; eight people living with dementia) were interviewed, and five themes identified: (1) Service issues; (2) Access issues; (3) Relying on own initiative; (4) New inequalities due to COVID-19; and (5) Missing out on the benefits of support services. Participants reported transport, finances, and location as factors reducing their ability to access support service pre-COVID, with inequalities remaining and at times exacerbated since. Carers and people living with dementia also reported struggling with accessing basic necessities during COVID, including food and medicines.

**Conclusions:**

Considering the benefits of accessing support services, resourced procedures and facilities are needed to maintain access to support services with more accessible remote support provision, enabling people from all backgrounds to access the care they need.

## Background

Affecting an estimated 50 million people worldwide [1], dementia is a global public health priority [2]. Within the next 10 years, the global number of cases is estimated to reach over 80 million [1]. Social support services provide vital care to people after a diagnosis of dementia, including for those caring for someone with it. Support services, offered after a diagnosis, vary and include out-of-home care such as day care centres and peer support groups. Services also include paid carers visiting the home and supporting the person living with dementia with activities such as medication management, cleaning, feeding, shopping, dressing, and toileting. Services are provided by different providers, ranging from local authorities to third sector organisations, and NHS Trusts occasionally. Accessing these types of services not only provides important social engagement with peers, but is also linked to improved well-being ([3]; Willis et al., 2018 [4, 5];). Moreover, they may provide essential temporary respite for unpaid carers, who are providing the greatest proportion of care for dementia in the UK (Alzheimer’s Society, 2014).

However, access to support services is unequally distributed. A growing body of evidence starts to identify inequalities at all stages of dementia care – ranging from inequalities of receiving a diagnosis in the first place [6] to inequalities in accessing post-diagnostic care, including anti-dementia medication [7–9]. Belonging to an ethnic minority group for example can be a barrier in using anti-dementia medication [10, 11], which is also a considerable difficulty for people from lower socio-economic backgrounds [12]. Geographical location also determines access to support services, with many people residing in rural regions experiencing limited to no access to formal care [13, 14]. People living with rare dementias can also face access difficulties. This is because most services are tailored to the needs of older people with the most common form of dementia, Alzheimer’s disease. People with young-onset dementia for example struggle finding suitable age-appropriate activities and peer support services [15]. People with other rarer forms of dementia, such as Lewy Body dementia, behavioural-variant fronto-temporal dementia, or posterior cortical atrophy, struggle due to their different symptomatology, such as increased behavioural problems, motor difficulties, or vision impairment [16, 17].

The COVID-19 pandemic and imposed public health restrictions in the UK since the 23rd of March, including social distancing measures and a nation-wide imposed lockdown, have made it impossible for most people with dementia and carers to continue receiving existing social support [18]. Some of the core benefits of social support services are the provision of personal social engagement as well as personal care [4, 19, 20]. In a recent Dutch study, social engagement was also found to be of importance in care home residents, highlighting the benefits of a simple easing in visiting rights for family carers during the pandemic [21]. Considering that social distancing measures and other forms of public health restrictions are staying in place for the foreseeable future, despite limited vaccination roll-outs, it is important to enable everyone, from whichever background, to be able to access adequate support. Inequalities in accessing remote support, where available, might thus be further exacerbated by a lack of access of digital technology in people from lower socio-economic backgrounds, and potential digital literacy issues [22]. A recent UK-wide COVID-19 social survey has highlighted that in the first 3 weeks of lockdown, people from more socio-economically disadvantaged backgrounds experienced more difficulties in accessing food and medication, compared to those from less disadvantaged backgrounds [23].

The aim of this study was to explore the experiences of accessing post-diagnostic dementia care for people living with dementia and carers both before and since the COVID-19 pandemic, and potential associated inequalities. It appears that no research to date has investigated this issue since the pandemic. Considering the importance and benefits of social engagement and social support services in dementia [21], it is important for everyone to receive the same opportunities in accessing the care they need. Findings from this study will provide important insights into the barriers of social support provision in dementia during the pandemic and post-COVID-19.

## Design and methods

### Participants and recruitment

Unpaid carers who were caring for someone living with dementia at the time of the study or those who have cared for a person living with dementia in the past, and where still accessing social care or social support services such as peer support groups, were eligible to take part. Unpaid carers had to be aged 18 or over. People living with dementia with mental capacity were also eligible to take part.

Participants were recruited via convenience sampling via social care and social support services and third sector organisations predominantly across the North West Coast of England, but also across the UK via dementia support organisations. For this purpose, services provided information about the study in regular newsletters, social media channels, and directly contacted eligible participants over the telephone and discuss the study. Contact details of interested unpaid carers and people living with dementia were forwarded to a researcher, who then contacted the participants.

We obtained ethical approval through the University of Liverpool [ID 7626].

### Data collection

Telephone interviews took place during April 2020, which lasted between 10 and 125 min (mean 27 +/− 19 min), and were audio-recorded. At the beginning of the interview, the researcher took verbal informed consent, which was also audio-recorded. Interviews were conducted by CG, KH, and trainees of the University of Liverpool Doctorate in Clinical Psychology course, all of whom were experienced in conducting qualitative research. The semi-structured interviews were conducted using a topic guide, containing questions about the participant’s service use before and after the COVID-19 outbreak and governmental restrictions.

Our topic guide was co-produced between the academic team, practitioners, people living with dementia and carers, focusing on experiences before and after the COVID-19 pandemic in respect of care services, coping, symptoms, challenges, benefits, strategies and impacts. The topic guide is published elsewhere [24].

Interviews were transcribed by a professional transcriber from the University of Liverpool, and in the process anonymised.

### Data analysis

Interview data were analysed using both inductive and deductive thematic analysis (Braun & Clarke, 2006) by eight research team members (CG, MG, SB, LS, JC, SC, KH, KW) and trainee clinical psychologists. Each transcript was coded by hand by two researchers/trainees individually, and identified codes were discussed jointly to generate themes. Thirty-five transcripts were initially coded using inductive thematic analysis, which were discussed between researchers. This generated the identified themes, with subsequent transcripts being coded using deductive thematic analysis based on the originally derived themes to complement those themes.

### Public involvement

One person living with dementia and three (former) unpaid carers formed part of the research team, and assisted with conceptualising the study, designing the interview guide, interpreting the findings and with dissemination. This ensured that the study and the interpretation and implications of findings were grounded in the lived experiences of those affected by dementia. The person living with dementia public adviser received a fee for each activity, such as reading through study documents or drafting a lay summary, according to NIHR INVOLVE guidelines (2005). The three carer public advisers were also running social support services, so were involved on that basis.

## Results

Fifty people (42 carers and eight people living with dementia) participated in this study. Most participants were female (*n* = 38, 76%), with most carers being spouses (*n* = 23, 55%). The majority of carers were living with the people living with dementia, with five carers where the people living with dementia resided in a care home. Carers were on average 60 (+/− 9) years old. The types of dementia varied from Alzheimer’s disease dementia (*n* = 21, 43%) to Lewy body dementia (*n* = 3, 6%) and vascular dementia (*n* = 8, 16%), with 12% (*n* = 6) of unpaid carers caring for/ people living with dementia living with young-onset dementia without a specific dementia subtype. The average time since the dementia diagnosis was 5 (+/− 3.6) years. Carers lived in a mix of disadvantaged and more affluent neighbourhoods, based on their IMD Quintile, with people living with dementia tending to live in more disadvantaged neighbourhoods (see Table 1). For more demographic details please see Giebel et al. [10].

**Table 1:** Index of Multiple Deprivation for carers and people living with dementia

IMD Quintile	Carers (***n*** = 42) N(%)	People living with dementia (***n*** = 8) N(%)
1 (least disadvantaged)	5 (13.2%)	0
2	14 (36.8%)	0
3	8 (21.1%)	1 (14.3%)
4	4 (10.5%)	3 (42.9%)
5 (most disadvantaged)	7 (18.4%)	3 (42.9%)

Five overarching themes were identified: (1) Service issues; (2) Access issues; (3) Relying on own initiative; (4) New inequalities due to COVID-19; and (5) Missing out on the benefits of support services. Table 2 shows the coding tree of the resulting themes and subthemes.

**Table 2:** Coding tree following thematic analysis

Themes	Subthemes
Service issues	• (Un)suitability of the support • (Un)suitability of adapted services
Access issues	• Lack of guidance and difficulty accessing support • Postcode-lottery/social inequalities • Associated costs • Transport
Relying on own initiative	• Dependency on carer motivation
New inequalities due to COVID-19	• Poor access to essential care and basic needs
Missing out on benefits of support services	• From the person with dementia’s perspective • From the carer’s perspective

### THEME 1: Service issues

#### (Un)suitability of the support

Where dementia support was identified for some people living with dementia, further issues were noted in terms of suitability. For some, the available support groups did not appeal to their interests, whilst particular groups felt the available support was too generic and did not meet the needs of the wider dementia population.

*“I’m not really a person that needs to be with a group and to be chatting and talking and laughing, I’m quite more just want to read and learn stuff”* ***Female person living with dementia, 67 years old***

People living with or caring for someone with young-onset dementia were subject to barriers in accessing and using care, as day care centres and group activities were deemed by many to be unsuitable. The respondents did not feel that there was clear information provided to aid identifying suitable support that met their needs. This resulted in a lengthy process of trial-and-error, sampling various support groups until a suitable one could be found, and further delaying the benefits gained from accessing support after initial diagnosis.

*“I've got to say that I had to take a proactive approach to find them [support services] as opposed to people offering me suggestions, service provision to me has been practically zero. Everything that I do, and I have done a lot, but it is down to word of mouth as opposed to anything that’s structured”* ***Male person living with dementia, 61 years old***

#### (Un)suitability of adapted services

Respondents recognised that a few dementia services attempted to adapt to the COVID-19 restrictions by offering alternative forms of support, such as video calls and online-led activities. However, for many, the online nature of these adapted forms of support were deemed unsuitable and exclusive to those with internet and access to an electronic device (which was often not the case for people living with dementia). Where adapted forms of support could be accessed at home by the people living with dementia, it was frequently discussed that these adapted services were of lower quality and did not compare to face-to-face contact.

*“I mean the lady that I help run Moving Memories with, she's a yoga teacher so we used to do armchair yoga, so she's put that online for us, so we can access that, but again it’s only available to the people that have the internet.”* ***Female carer (daughter), 60 years old***

*“it lightens our day, we love it, we love our friends, our friends are our life these days because life is not the same once dementia hits, it’s a different group of friends and they are our real friends and we miss them, we really really do miss it all. But having our What's app group has meant that we have been kept in touch which, if this had all struck, five or six years ago we would be stuck wouldn’t we, we wouldn't have what we've got now so we are really lucky that we've got the What's app but we really do miss the personal contact with our friends”* ***Female carer (spouse), 62 years old***

### THEME 2: Access issues

#### Lack of guidance and difficulty accessing support

Access to dementia support services before the time of COVID-19 was mostly described as a difficult and challenging process. Long waiting lists and poor communication between the various services and people living with dementia led to delays in accessing support. The impact on delayed access to support was described by fears of long-term social isolation.

*“we did eventually manage to access our Link Worker, it took another twelve months after his diagnosis to get that. I've been fighting to get some support for his meals since September 2019 and we are about to get the support just before Christmas when there was a change in the occupational therapist and then of course we had the Christmas rush and he put on to the waiting list... my father was again on the waiting list to go and join a day centre with him, we are extremely worried and concerned about him becoming socially isolated”* ***Female carer (spouse), 52 years old***

*“Within the past year probably we’ve been engaging with Social Services to try and engage my Dad and get him some support and support for myself and my aunt but that’s been a slow process just trying to you know build up trust in relationships and assess how that’s going to work”* ***Female carer (daughter), 40 years old***

Many respondents recounted a lack of clear information provision in the early stages of the dementia diagnosis, to support them in identifying suitable support services, or even locating the necessary funds to allow them access. This uncertainty led to high levels of stress and dependency on informal peer support groups, as unpaid carers and people living with dementia relied on shared experiences to guide them in locating support.

*“services exist but it is hard work finding what is out there and unless you are fully in the system and in a memory service in the residential address it is almost impossible to access information as to what’s out there”* ***Female carer (daughter), 56 years old***

*“we wanted a list of you know [support options] rather than people have to go looking for what's available, it would be given to them 'cause from day 1 you should have that information in front of you, not have to go looking for it, or hear it through the grapevine, or having someone to talk to like you know a piece of paper is not the same as having someone to talk to”* ***Male person living with dementia, 67 years old***

#### Postcode lottery/social inequalities

Those paying for care and support recognised differences between themselves and other people living with dementia in terms of funded access to local services. Differences in receiving funded support reportedly varied dependent on the financial abilities of the local councils, thus furthering themes of a ‘postcode lottery’ and social inequalities. Postcode lottery is a frequent term used in the UK referring to people living in different postcodes within a city for example being able to access different services, purely based on which clinical Trust or local authority covers a particular postcode. This can result in people living in certain postcode receiving free social care services, whereas others who may live one street away, yet are in a different postcode catchment area, are served by a different clinical Trust or local authority, which do not cover these costs. This resulted in feelings of anger and frustration towards those receiving funded support, and the organisations or local councils that have had to withdraw support in their local area.

*“we need more places like that [day centres] but I have to pay for this, after working and my husband worked, he’s 82, I’m 70 nearly 75, we’ve worked since we were 16 and we have to pay for this and yet people who go to these the Council have closed down everything because there’s no funding for them”* ***Female carer (spouse), 74 years old***

*“the [charity organisation], they were running the singing group originally and when they abandoned Liverpool three of us decided we [cannot] let it go so we kept it going”* ***Male carer (spouse), 71 years old***

#### Associated costs (unequal variation between people/areas)

High costs were frequently associated with a range of the day centre and support groups activities for people living with dementia, and this featured as a key topic in the discussion of accessing support. High costs were viewed as a barrier to obtaining support, but one that must be factored into their everyday lives as an essential need for people living with dementia and their carers.

*“no nothings for free… golf sessions well it’s about 100 pounds a month and that’s for 3 lessons in that month, the Dementia Forward we pay 20 pounds for the day with the with the group but they’ve just recently got some funding from the lottery so I think in the future for the next months or so it will be free but obviously it’s not happening at the moment [since lockdown]so there’s never there’s not been anything that we’ve had for free no”* ***Female carer (spouse), 58 years old***

High costs were also discussed in terms of care home fees for people living with dementia. Again, this was seen as an unfair variance between those that could afford to pay (but would struggle) and those that met the criteria for a funded place.

*“I don’t want to put him in a home but if I did we would not come under the criteria of the £23,000 I would have to pay, I would say at least a thousand pounds a week, how that can be justified I do not know. But we would have to pay a thousand pounds a week for my husband to go and knowing full well that there could be a person sitting alongside him who is being funded by the council and partially funded by us as well because they are subsidised by what we pay”* ***Female carer (spouse), 74 years old***

*“We were very very careful who we chose mum is among the 60% of people that has to pay for her care and obviously costs vary wildly so we knew what mums budget would be and that sort of excluded a certain number of care homes because it was so wildly expensive.”* ***Female carer (daughter), 56 years old***

Contrasting reports of easy access to services were noted by those people living with dementia that met the criteria for funded care, or due to the financial capabilities of the local council. However, even where the services or activities were free of charge for these people living with dementia, the associated costs of food and travel had to be considered.

*“the day care centre is funded through direct payments from the Council so they fund 3 days. It was originally 1 day a week when she went and then we’ve increased it to 2 and then increased it to 3 and they do fund all of that. The Council also fund the day to day care the personal care in the morning, the sit ins are I use the sit in vouchers the carers vouchers which come from City Council so we get 5 of those a week so yes the carers most of the care is funded by [area name] City Council”* ***Male carer (son), 54 years old***

*“we were paying for his meals and they were they were going to start charging for bringing back on a on a you know they would drop him after the club had finished so they were they were going to start charging for that”* ***Male carer (spouse), 71 years old***

Few reported a lack of need for additional dementia-specific support, which appeared to correlate with accounts of mild/manageable forms of the dementia condition, having a comfortable living space, access to a garden, and being financially secure. This was however viewed as unusual in the eyes of the unpaid carer.

*“I’m sort of half not embarrassed at saying this it’s just a fact. We are very comfortably off, we have a big house, a nice garden we were not reliant on any social care, she is physically mobile she is not aggressive or anxious or wonders or tries to do things”* ***Male carer (spouse), 68 years old***

Therefore, those people living with dementia that sat in the middle of the socioeconomic ladder, who neither met the criteria for funded care nor felt financially able to support themselves, reported feelings of unfair inequalities to accessing dementia support, signifying a social health inequality.

*“we haven’t wasted our money we’re not rich, we’re not wealthy but we’re just comfortable and because we’ve done that and we haven’t smoked and drank our money we’re being penalised”* ***Female carer (spouse), 74 years old***

#### Transport difficulties

Where people were accessing face-to-face services, transport was often noted as a concern. Services can be geographically difficult to access especially for those with no car, with taxis often being too expensive for frequent service visits. Using public transport can also pose its difficulties for people living with dementia, with many people living with dementia being not independent enough to seek out the right bus and follow the timetable, and as a result lack access to services.

*“there’s no, the access, there’s access to services and things if you can get there. Well if you’ve got dementia you can’t always get there my mum’s lost all confidence on being able to catch a bus herself and that’s been for 2 years so she’s not been independent at all”* ***Female carer (daughter), 48 years old***

### THEME 3: Relying on own initiative

The respondent accounts portrayed the need to rely on the motivation of the unpaid carers, to secure the most suitable dementia support available, before COVID-19 and especially during the pandemic. This furthered themes of lack of clear information provision and the unsuitability of some of the support offered.

*“you don’t need to be told to go off to this place where they do singing, my husband he wouldn’t like anything like that because he won’t cooperate and I do have a lady at the [charity organisation] but she’s got in touch with this over the virus but I haven’t heard from her she never rings up and says Kath how are you coping [during COVID-19].”* ***Female carer (spouse), 74 years old***

Where support was accessed effectively, this was often due to the unpaid carer’s proactivity. This may be perceived as a social/health inequality, as those people living with dementia who do not have a supportive network of cares to actively identify support, would not be able to benefit from the available services.

*“I gave up my work in 2016 because we realised both my parents were not coping and so I gave up [work] in order to spend a day or 2 a week with them, driving up from where I live near {area name}, up an hour and a half to help… that meant going to various appointments with mum, her consultants appointments, but I always wondered how people coped if they were on their own because it seemed to me that I was doing an awful lot of sort of, I was like their admin person really.”* ***Female carer (daughter), 58 years old***

Unpaid carers frequently reported making sacrifices to their own lives to effectively care for the people living with dementia, furthering the notion that carers must possess a proactive and motivated nature in order to carry out this role. Such sacrifices included moving house, giving up employment and travelling far distances to ensure the people living with dementia is cared for.

*“my brother has had to move in, he's had to, 'cause he's been working from home for his work he's had to move his home office in with my father just to help my father with meals and with prevention of contamination as much as we can”* ***Female carer (spouse), 52 years old***

### THEME 4: New inequalities due to COVID-19

People living with dementia were not put on the government’s vulnerable list because of their dementia as such, but only of they had an underlying health condition. Thus, they were not offered priority slots for grocery shopping or offered additional support and advice from the government despite the perils of living with dementia. Obtaining food and medical supplies was therefore a struggle for many during the pandemic. Where those people living with dementia were given access to priority support, it was reportedly due to other health comorbidities and not the dementia.

*“so I made a conscious effort of doing the shop down at [supermarket] and then going to coming back and going straight online to do to get the next one but didn’t think about going in and doing one after that that’s when I stressed myself out because I couldn’t get another slot and then not getting a letter and not having proof until last week that I am classed as highly vulnerable that that stressed me out as well so you know I don’t think it’s been straightforward”* ***Female carer (spouse), 61 years old***

*“I’m in the vulnerable group because I've got heart disease and COPD and erm but also living with cancer”* ***Male person living with dementia, 61 years old***

Where the person living with dementia was classed as vulnerable, for reasons other than the dementia, they faced further barriers to accessing the support and medical services they needed, due to the novel corona virus health risks and the social restrictions on leaving the home to attend a health clinic.

*“we tried ringing the doc, I’d already spoken to the doctor and said the medication that she’s [person living with dementia] on doesn’t seem to be keeping her calm or anything like that could we change it and they were reluctant to do that, [the doctor] referred us to the mental health team, when I spoke to them they then decided that she was, because of the coronavirus she was in a vulnerable group, just then signed us off the service my sister rang the doctors to ask them what what’s the best thing and to be honest we ended up in Accident & Emergency.”* ***Female carer (daughter), 48 years old***

Further fears and anxieties were expressed in terms of accessing basic health services during the time of the COVID-19 pandemic. Various community health practices were unavailable to the people living with dementia and/or their carers due to the limited capabilities of the health care providers, and the participants discussed fears that closures may impact on their health in the meantime.

*“services in hospitals such as dentists, I've no access to that at all, meetings have been cancelled. Wheelchairs that’s put on-hold. OTs they, I have had a little bit of help from OTs but generally the services that were available [on the] NHS seem to have been withdrawn virtually completely. Or put in limbo, I haven’t suffered as such but there are items such as dentistry. [Wife with dementia] has no teeth or the teeth that she's got are in an extremely bad state, I would hate anything to happen there because I don't know what we would do. But it seems as I said services are withdrawn, doctors are on the phone, everything’s in limbo”* ***Male carer (spouse), 69 years old***

### THEME 5: Missing out on benefits of support services

#### From the person living with dementia’s perspective

The respondents frequently discussed the benefits of receiving support or attending support services before the time of COVID-19. Such benefits were twofold, benefitting both the people living with dementia and the unpaid carer. The people living with dementia benefitted from socialisation, keeping fit and active, and peer support, all of which were lost in the face of COVID-19 due to closure of support services. The lack of routine and familiarity visibly impacted on the health and wellbeing of the people living with dementia, in terms of loss of cognition, loss of motivation, memory loss and confusion.

*“we found if [person living with dementia] don't see anybody regular and they don't have that meet up it really, really puts them out… it’s devastating, it really is. Most of the time now my dad just wants to sleep. So he's gone from being very active to wants to sleep. We facetime each other and others in the group you know a couple of times… we've had a zoom conference this morning and my dad could hardly recognise them. It’s like he's lost that contact, he's lost that focus. And I'm just wondering whether we will get that back at the end of it all.”* ***Female carer (daughter), 60 years old***

#### From the carer’s perspective

Unpaid carers benefitted from respite and independence whilst people living with dementia attended support groups and day care centres. It was apparent that the unpaid carers formed a community and informal peer support through shared understanding and experiences in caring for people living with dementia. This provided additional benefits as they were able to navigate the range of activities available. The cessation of support groups during the time of COVID-19, subsequently resulted in a loss of support, as well as loss of respite and downtime for the unpaid carers.

*“when we go to the [charity organisation] cafes, there's always somebody from the [organisation] there present so if you’ve got a query you can ask, but it’s also nice at these sessions as well where the carers can talk to other carers and share their experiences while, like my dad and the others, you know chat about the old days. So it’s a bit of respite for both of us really”* ***Female carer (daughter), 60 years old***

*“obviously as the times gone on [since lockdown] there are still things that need doing and I’m trying to do that alongside caring for him [husband with dementia] and I’m becoming very aware of there being absolutely no downtime”* ***Female carer (spouse), 60 years old***

## Discussion

This is the first study to have explored inequalities in dementia care service access before and since the COVID-19 pandemic. Previous inequalities in accessing support services appear to have been exacerbated since the pandemic, highlighting the urgent need for more targeted support to enable equitable access for people living with dementia and carers from any background.

Corroborating growing evidence, both carers and people living with dementia reported various issues with accessing support services before the pandemic, including financial issues and where they lived, amongst others [7, 11]. A great deal of previous literature that has explored inequalities in dementia care is based on quantitative data, often employing routine or cohort data to show how people from socio-economically more disadvantaged backgrounds or from minority ethnic groups experience reduced service utilisation (i.e. [25, 26]). Qualitative research to date has mostly explored specific types of inequalities, such as rurality [14] and ethnicity [27], whilst findings from our study expand the evidence base showcasing a multitude of pre-COVID-19 inequalities. Specifically, these inequalities appear to have been exacerbated by the pandemic, raising concerns for the need of improved clinical and social care support, such as enabling improved accessed to care regardless of postcode, which might involve improved digital support to allow remote care. This is particularly relevant and topical given the continued social distancing measures which will remain in place throughout this pandemic.

Inequalities in accessing dementia care reportedly coalesced with area deprivation and available funding support from local councils, as well as personal costs associated with obtaining care. Such findings have been reported previously, with most evidence linking the lowest socioeconomic groups with poor access to health services, due to factors including cost of paying for healthcare, lower educational attainment, and transport (Bradford et al., 2009 [28, 29];). Those people living with dementia and carers who sat in the middle of the socioeconomic ladder, who neither met the criteria for funded care nor felt financially able to support themselves, reported feelings of unfair inequalities accessing dementia support, signifying a social health inequality. To date, this has not been evidenced for any area of dementia care, highlighting a significant inequality in access to dementia care causing detrimental effects to the lives of those affected by dementia. Generally, despite free healthcare nationally for UK residents, similar inequalities have been noted when accessing a number of health services which remain fee-paying (Shaban, 2003 [30];). However, those in the lowest socioeconomic groups and those with health risk factors are eligible for state-funded care, with research suggesting that exemptions of co-payments for certain groups contributes to inequalities in accessing health services [30]. This resultant group, described in previous literature as “the squeezed middle-class”, has been recognised as struggling in terms of financial support and subsequent access to paid services [31], which supports our present findings on those people living with dementia and carers in the middle of the socio-economic ladder. However, it should not be overlooked that people living with dementia in the lowest socioeconomic classes in the UK face greater inequalities overall [12, 32]. Moreover, where fully funded support was offered to the participants of this study, they reported additional costs incurred through transport and meals, thus no support was ever completely free of charge.

Poor access to basic necessities since the pandemic, such as food and medication, was also reported by both people living with dementia and carers. This not only posed an inequality, but a potential threat to the health and wellbeing of people living with dementia. Similar findings have been reported in the general (non-dementia) UK population, whereby basic food and medical care have been difficult to obtain for some during the pandemic [23]. However, the study related this finding to financial stressors, namely loss of income, which does not explain the finding from the present study. Instead, participants assigned the lack of access to such necessities to missing out on online shopping slots due people living with dementia being overlooked as a vulnerable group for example, as well as the practical issues of shopping with/for a people living with dementia during the time of COVID-19. This suggests clear practical access issues, in parts due to non-recognition of dementia as a vulnerable group by the government, highlighting the need for improved support for people living with dementia not only in terms of care during the pandemic, but also in terms of supporting easy and equitable access to basic necessities.

Receiving access to support services does not solely depend on someone’s financial background or geographical living location though. In most instances, proactive carers are finding out about support groups and day care centres for example by searching the internet or by contacting service providers. What should happen is that people living with dementia and carers receive information about different local services after a diagnosis and during a post-diagnostic support group, which is only sometimes provided to people depending on their NHS Trust. However, the it appears that many people receive little to no information about services, but only information about the diagnosis, and often lack a single contact person for questions about the dementia and continued support despite official recommendations [33]. This could be an admiral nurse for example or a link worker. One possible solution would be to increase the number of Admiral Nurses in the UK, or generally provide a link contact who is checking in with the people living with dementia and the carer on a regular basis [34, 35]. More importantly, some people living with dementia do not have a carer, and if accessing vital social support is mostly based on having a proactive carer, then that leaves people living with dementia without an unpaid carer at an even greater disadvantage to accessing care.

Whilst this study benefits from a large sample capturing the experiences of those affected by dementia shortly after lockdown, this study is subject to limitations. Participants were mostly from a White ethnic background, and thus potential inequalities specific to ethnic minority status were not fully captured. Many ethnic minority groups face barriers to accessing mental health and dementia care services for example [36, 37]. COVID-19 is likely to have led to further barriers, which have not been captured in our study. Future research needs to explore access issues in people from minority ethnic backgrounds since COVID-19 in more detail, particularly in the light of their increased COVID-19 infection-related risks [38]. Furthermore, people with dementia may potentially have had a recall bias thinking back to their experiences of services pre-COVID, due to their cognitive impairment. However, we asked about their experiences and not about specific number of times they accessed services for example, which might be more difficult to remember. In addition, interviews were conducted within up to 6 weeks since lockdown, so not very long after the ‘pre-COVID’ time.

## Conclusions

This is the first study highlighting access issues and experiences of social support service usage for dementia both before and since COVID-19, indicating various inequalities in accessing services in both time periods. With the second UK lockdown over, and vaccination only started being made available to limited groups of the population, the pandemic is expected to stay and continue affecting people’s lives in the near future, having already led to 10,000 more dementia deaths in the UK than normally (ONS, 2020). Thus, our study highlights the need for services to be adapted to continue providing support and particularly enable easier access for people from any background. This would require greater government support into the struggling social care sector, so that people are not faced with paying for day care or home care when they face difficulties in affording basic necessities even, or are on the middle of the socio-economic ladder. Considering a general growing need for social care for dementia over the coming years [39], and that COVID-19 is linked to dementia-like syndromes [40], this study highlights the need for greater support into removing inequalities in dementia care, with inequalities newly emerging during the pandemic and remaining post pandemic.

## Data Availability

The qualitative dataset generated and analysed during the current study is not publicly available due to ethics, but is available from the corresponding author on reasonable request.
